# “That Was an Eye Opener for Me”: Mixed-Methods Outcomes Educating Texas Community Health Workers on HPV Vaccination Using Project ECHO^®^

**DOI:** 10.3390/vaccines12070806

**Published:** 2024-07-20

**Authors:** Shaylen Foley, Ashleigh Flowers, Tralisa Hall, Matthew T. Jansen, Michelle Burcin

**Affiliations:** 1 Interventions and Implementation Department, American Cancer Society, 3380 Chastain Meadows Pkwy NW, Suite 20, Kennesaw, GA 30144, USA; ashleigh.hayward@cancer.org (A.F.); michelle.burcin@cancer.org (M.B.); 2 Independent Researcher, Chapel Hill, NC 27514, USA

**Keywords:** HPV vaccination, evaluation, community health workers, Project ECHO, education, Texas, knowledge, confidence, beliefs

## Abstract

Human papillomavirus (HPV) is known to cause six different types of cancer. HPV vaccination can prevent over 90% of these cancers. Community health workers (CHWs) have the potential to drive HPV vaccination demand through education and navigation by addressing vaccine hesitancy and dis/misinformation and by reaching non-English speaking, vulnerable, or rural populations. Despite their possible reach, there is limited research on HPV vaccination education programs for CHWs. In 2020–2021, the American Cancer Society (ACS) HPV Cancer Free Texas (HPVCFT) Project implemented the eight-session Mission: HPVCFT Vaccination ECHO–CHW Program ten times. This manuscript details the program’s implementation processes and outcomes. The program used the Project ECHO model and was offered in both English and Spanish. One hundred and forty-six Texan CHWs completed pre- and post-training surveys. The participants demonstrated significant HPV vaccination knowledge increases and desirable shifts in their foundational HPV vaccination beliefs, including the belief that the HPV vaccine is for cancer prevention. The participants also reported increased confidence in communicating about the HPV vaccine in the community. Improving knowledge, beliefs, and confidence in HPV vaccination is the first step in addressing concerns and increasing uptake. Future research and interventions are needed to better understand how CHWs can be more systematically linked to vaccination opportunities or provided with clearer paths for directing patients to providers that vaccinate.

## 1. Introduction

Human papillomavirus (HPV) is known to cause six different types of cancer, including cervical, vaginal, vulvar, penile, anal, and oropharyngeal. It is estimated that approximately 36,500 HPV-related cancers occur in the U.S. each year [1]. HPV vaccination can prevent over 90% of these cancers [2]. The American Cancer Society (ACS) recommends routine vaccination between ages 9 and 12 [3].

Despite the benefits of the vaccine, only about 50% of U.S. adolescents aged 13 were up to date on HPV vaccination in 2021 [4]. Additionally, the COVID-19 pandemic resulted in a deficit of 3.9 million (−13.6%) HPV vaccine doses ordered, with greater disparities seen in rural areas and publicly funded doses, highlighting a current need for adaptive programming that can reach these populations [5,6]. The ACS has been committed to increasing HPV vaccination uptake and education since 2015 [7,8,9,10,11]. The HPV Cancer Free Texas (HPVCFT) Project aims to increase HPV vaccination completion rates in Texas from 40% in 2017 to 80% in 2026. One tactic that the ACS HPVCFT uses is to educate and mobilize community health workers (CHWs) on HPV vaccination. 

A CHW (also referred to as a promotora) is a public health professional who works in communities to increase knowledge on various health topics and helps to connect community members to health and social services [12]. Research shows that CHWs have a positive impact on the social determinants of health in their community. They provide health education, link members to health resources, and advocate for patients and community members [13,14]. CHWs are valuable in addressing health inequities and increasing access to care for rural communities and vulnerable populations [13,14,15,16,17,18,19]. Often, the focus of their work is chronic disease management, such as diabetes, cancer, asthma, and heart disease [13,15,17,18,20,21,22]. There is minimal research on CHWs and HPV vaccination in the U.S. [23,24,25].

The state of Texas lags behind most of the country in HPV vaccination coverage, making it an ideal place to focus on uptake [26]. Texas has a Promotor(a) Training and Certification Program managed by the Texas Department of State Health Services (DSHS). The program provides training, certification, and continuing education. In 2021, there were a total of 4208 certified CHWs in Texas, the largest number of any U.S. state [12]. 

To reach CHWs across Texas, ACS uses virtual education via Project ECHO (Extension for Community Healthcare Outcomes). Project ECHO presents a novel tele-mentoring initiative designed to address healthcare disparities and enhance medical access in underserved communities [27]. It uses a hub and spoke model to spread knowledge virtually from and between subject matter experts and participants to improve health outcomes. It also encourages case-based learning. There have been several Project ECHO programs implemented with CHWs in the U.S., however, few studies have looked at Project ECHO to educate CHWs on HPV vaccination [28,29,30,31]. The focus has been primarily on cervical cancer screening, diagnosis, and treatment [32,33].

Overall, there is limited research on HPV vaccination education with CHWs. The state of Texas has a low HPV vaccination coverage, is highly populated, and has a formalized state CHW program for certification and continuing education, making it an ideal setting for targeting CHW knowledge and education on HPV vaccination. In this paper, we describe the program and outcomes of the virtual *Mission: HPV Cancer Free Texas Vaccination ECHO—Community Health Worker Program* using the Project ECHO model.

## 2. Materials and Methods

In 2020, we developed a virtual 8-session education program in English and Spanish on HPV vaccination for CHWs in Texas using the Project ECHO platform and model. This 8-session program was implemented 10 separate times between 2020 and 2021. This paper presents quantitative and qualitative evaluation results following the first two years of implementation.

### 2.1. Educational Objectives

The goal of this ECHO was to educate CHWs on their role in HPV vaccination, to increase positive attitudes and beliefs about the HPV vaccine, to increase CHWs’ confidence in advocating for the HPV vaccine in their community, and to equip CHWs with tools and resources.

### 2.2. Participants

CHWs were recruited through Texas DSHS announcements using a convenience sample. Participants qualified if they had a CHW certification or were becoming certified, spoke English or Spanish, and lived in Texas. The participants received 1.5 h of Texas DSHS Certified Continuing Education (CE) credits for each session attended. Those that attended all 8 sessions could receive up to 12 CE credits.

### 2.3. Course Structure

The 8-session program ran for around 4 months, with sessions every other week at approximately the same day and time. English and Spanish programs ran concurrently with a consistent community health worker instructor (CHWI) and subject matter experts. In 2020, we hosted three English programs (English 1–3) and a single Spanish program (Spanish 1). In 2021, we hosted three English programs (English 4–6) and three Spanish programs (Spanish 2–4). 

### 2.4. Curriculum Content and Development

We adapted the ECHO model, typically for clinical audiences and topics, to CHWs. We partnered with the DSHS and Texas A&M Health Science Center to develop the curriculum and to obtain DSHS accreditation. We modified or used existing ACS HPV vaccination tools, resources, and messaging where appropriate. All content was developed in English and translated into Spanish. The session topics and competencies were as follows:

1. Role of CHWs in Immunization: the role of CHWs in the healthcare workforce, removing barriers, interacting with everyone, and being an immunization champion;
2. CHW HPV 101: the HPV vaccine as cancer prevention, HPV cancer statistics, the effectiveness of the vaccine, the recommended ages and doses of the HPV vaccine, and the importance of the vaccine;
3. Cultural Humility and Social Determinants of Health: cultural humility and sensitivity, service coordination, and community impacts such as reverse disparities and underserved populations;
4. Communicating with Parents: talking about HPV vaccination through provided strategies and approaches to communicate with parents about the vaccine;
5. HPV Myths: HPV facts to counter myths and HPV vaccine school requirements;
6. Debunking HPV Myths: how to address parent concerns, how to redirect questions, and what to do in different situations;
7. Tools and Resources: HPV tools and resources and strategies to find reliable sources;
8. Training Trivia: test on the material covered in the prior seven modules.

### 2.5. Session Components

Each session was 90 min and hosted live on ZOOM [34]. During each session, the instructor presented didactic content utilizing whiteboards, surveys, polls, and quizzes to encourage engagement and learning retention. The participants then presented an experience discussing the HPV vaccine. One to three case presentations were shared each session. The attendees and SMEs provided feedback to facilitate individual and shared learning. 

### 2.6. Evaluation Theoretical Framework

The program evaluation followed the CDC Framework for Evaluation in Public Health [35]. We mapped the objectives, implementation components, and needs of the participants. We developed an evaluation planning matrix (EPM) with questions, measures, data sources, and domains (Table 1). The Morehouse School of Medicine designated this evaluation as research not involving human subjects (protocol 2047442-1).

### 2.7. Quantitative Data Collection

The participating CHWs completed two online surveys over the course of the program: a pre-training survey that was sent to all enrolled individuals who attended an initial orientation session, and a post-training survey that was shared with participants during the eighth session and then sent out afterwards to all participants who had attended at least one session. We collected the survey data with SurveyMonkey [36].

### 2.8. Quantitative Measures

The pre- and post-surveys assessed HPV vaccination knowledge, beliefs, and confidence in discussing the vaccine. Knowledge was assessed using four items (Table 1). We assessed vaccination beliefs (three items) and confidence (one item) using a 5-point response scale ranging from strongly agree to strongly disagree. The pre-survey also assessed personal and professional demographics. The post-survey assessed program satisfaction (six items). Knowledge, questions, and confidence and belief statements were adapted from existing surveys which were modified to fit the content, goals, and audience of the ECHO [37].

### 2.9. Quantitative Data Analysis

Our analysis sought to understand the immediate outcomes of the ECHO training, comparing English and Spanish participants. A total of 796 individuals completed the pre-survey during or after the orientation session (653 were not duplicates). Not all who attended orientation joined the educational course; 750 individuals attended at least a single session. During the four-month course (approx. 120 days), we observed drops in attendance between the first and eighth session, with most attrition occurring after session one and two. A total of 249 discrete individuals attended the eighth and final session and 241 (226 were not duplicates) completed a post-survey. We matched the pre-survey and post-survey respondents using the following algorithm: first by email address, then by DSHS CHW ID number, and then by name. The process was stepwise, and after matching on a variable, if multiple matches were found, we kept only the most complete response that met our inclusion criteria. We excluded submissions with a pre-survey to post-survey timestamp gap of less than 0 or greater than 180 days (the median response gap was 111 days and the maximum retained was 134 days). At each step, we also excluded duplicate emails, CHW numbers, and names among matched and unmatched responses to prevent rematching responses in the next step using a different variable. After exclusions, we matched 146 responses, including 92 English and 54 Spanish participants. Of the 95 unmatched post-survey responses, exclusions were made for the following reasons: 69 failed to find a match (largely due to inconsistent name entry), 15 were duplicates, 4 had timestamps gaps outside 0–135 days, 3 had a pre- and post-survey in discordant languages, and 4 had both a timestamp or language issue.

We coded knowledge questions as correct or incorrect and tallied the total correct answers. We used an exact McNemar test to assess if the respondents improved after the program overall and on individual questions [38]. To assess the shifts in vaccination beliefs and confidence, we used collapsed McNemar tests. We consolidated the five-point scale into a two-point scale of “Desirable” or “Undesirable” based on the direction we desired respondents to shift in. We grouped Agree and Strongly Agree as “Desirable” for statements F1–F3 in Table 1. We grouped Strongly Disagree and Disagree as “Desirable” for F4 and F5. “Neither agree nor disagree” was classified for all statements as “Undesirable”.

We conducted analyses in R v. 4.211 and Stata v. 17 [39,40,41]. Additional R packages were used for data cleaning, tables, and visualization [42,43,44,45,46,47]. Comparable analytics were conducted on all non-duplicate responses at pre-training and post-training in a pooled approach using Pearson’s Chi-squared test with Yate’s continuity correction for the knowledge questions and the Cochran–Armitage test for the belief and confidence questions.

### 2.10. Qualitative Focus Groups

We facilitated three 60-min virtual focus groups in April 2022 with a convenience sample from the programs in 2020–2021. Each focus group contained three to five participants, for a total of thirteen individuals. The focus groups were conducted in English using semi-structured discussion guides and hosted on Microsoft Teams. The discussion explored general information on how CHWs engage in their communities and feedback on the program. We obtained verbal consent prior to starting video recording. Each participant received a USD 30 lunch gift card. 

### 2.11. Qualitative Data Analysis

We used Rev artificial intelligence to transcribe the focus group recordings and MAXQDA for analysis [48,49]. Our analysis combined an inductive and deductive approach. We generated a preliminary code list using the focus group guide. We updated the code list and definitions as new codes emerged. We grouped data by themes with descriptive text-based summaries and extracted corresponding quotes.

## 3. Results

### 3.1. Characteristics of Participating CHWs

In 2020–2021, the ACS implemented the eight-session *Mission: HPV Cancer Free Texas Vaccination ECHO—Community Health Worker Program* ten times, reaching 750 CHWs in Texas for at least one session and 350 for four or more sessions. The participants claimed a total of 4102 continuing education credits. A total of 146 pre- and post-survey responses were matched. 

Most participants were female (92.5%) and mid-career (mean age of 47.6, s.d. 12.6) (Table 2). A large portion reported secondary levels of education, including some college (21.9%), a bachelor’s degree (29.5%), or an advanced degree (11%). English CHWs most frequently reported having a bachelor’s degree (35.9%), while Spanish-speaking CHWs most frequently reported a high school or equivalent education (46.3%). A higher percentage of English participants reported working full time as a CHW (43.5%) compared to Spanish participants (18.5). A large proportion of all participants reported not being currently employed as a CHW (32.9%).

Of those who reported current or volunteer employment as a CHW (n = 98), more than half reported 5 or less years as a CHW. Spanish-speaking participants more often reported 10 plus years of experience (30.8%) compared to English participants (8.5%). Of the employed or volunteer CHWs, reported employment was at a non-profit organization (41.1%), healthcare agency (24.4%), university/academia (22.2%), social service entity (6.7%), or other (15.6%). Only 26.5% worked at an agency that vaccinated. 

The focus group participants held a range of job titles, years of experience, and responsibilities. They served highly varied communities, sometimes targeting highly specific populations, such as people living with HIV, refugee families, or cancer survivors. Others served in roles where they maintained their CHW qualifications but provided non-traditional CHW services, such as school nurses and professors.

*“…sometimes I work as a case manager as well. I wear multiple hats, and case management ensures that they [people living with HIV] get their medicine…”*

*“Normally, if I’m taking on a caseload, it’s gonna be a client who’s dealing with domestic violence, some legal issues, maybe some custody issues, and immigration issues. So there’s a lot of barriers to their qualifications for certain programs…”*

### 3.2. HPV Vaccination Knowledge Change

The participants demonstrated statistically significant increases in HPV vaccination knowledge, with more participants moving from incorrect to correct responses than the reverse between the pre- and post-surveys (exact McNemar odds ratio: 1.84, *p*-value: <0.0001).

**Figure 1:**
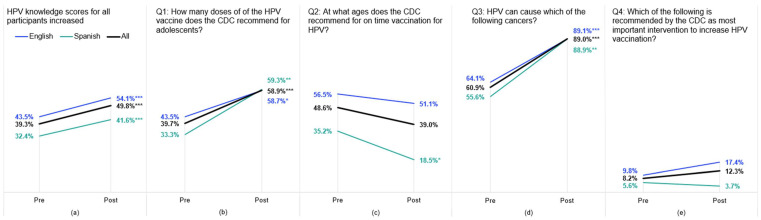
HPV knowledge scores pre- and post-ECHO training for **English**, **Spanish**, and **All** CHW participants 2020–2021: (**a**) total scores on all four HPV knowledge questions for English and Spanish participants and (**b**–**e**) average scores on each individual knowledge question disaggregated by English and Spanish cohorts. Significance derived from exact McNemar tests (*** *p* < 0.001, ** *p* < 0.01, and * *p* < 0.05).

The participants increased their average knowledge scores by 10.4 percentage points from a pre-score of 39.4% to a post-score of 49.8% (Figure 1). The English participants had higher average pre- and post-scores (43.5% to 54.1%) compared to the Spanish participants (32.4% to 42.6%), though both groups saw comparable increases in total correct responses.

Both the English and Spanish participants saw statistically significant increases (*p* < 0.001) for knowledge questions one and three on the recommended doses for the HPV vaccine and the cancers caused by HPV infections. The participants most frequently incorrectly answered question four about the importance of provider recommendations for HPV vaccination, with only 8.2% answering correctly pre-program and 12.3% post-program (a non-significant increase). While not statistically significant for all respondents, the participants were less likely to correctly answer question two about the recommended age for HPV vaccination post-program (39.0%) than pre-program (48.6%). Pooled analyses on all survey respondents, regardless of matching, found comparable results overall and by question for HPV knowledge change. 

We also asked the participants to assess their own knowledge of HPV vaccination pre- and post-program. We saw statistically significant shifts, with most participants prior to the program reporting “I know very little” (28.1%) or “I know some” (60.3%) and most reporting “I know a lot” (77.4%) or “I am an expert” (12.3%) post-program (*p* < 0.001).

### 3.3. HPV Vaccination Beliefs and Confidence

**Figure 2:**
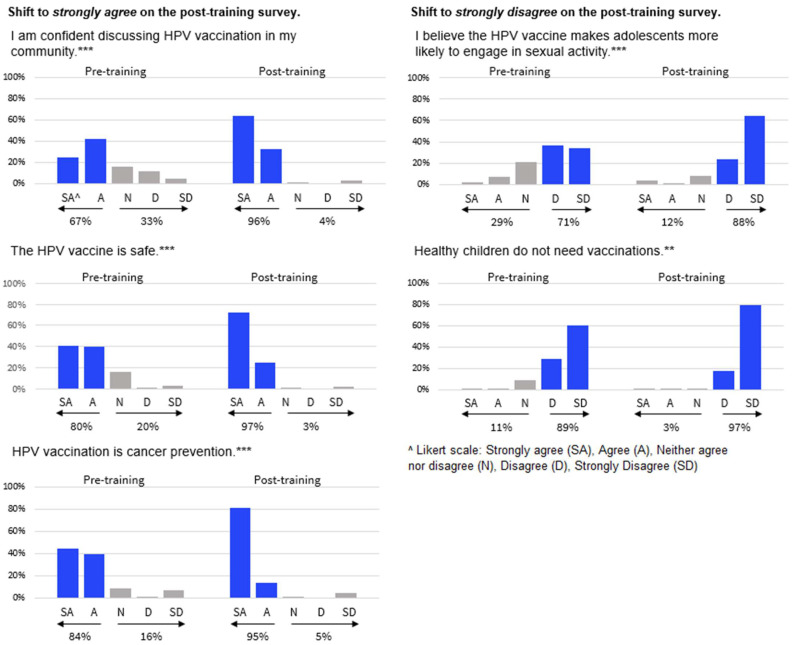
Changes in HPV vaccination beliefs and confidence among CHW ECHO participants (n = 146). Note: Significance derived from collapsed McNemar tests (*** *p* < 0.001 and ** *p* < 0.01).

The CHWs showed desirable shifts in their key HPV vaccination beliefs and confidence between pre- and post-program (Figure 2). Pre-program, 67% of the participants strongly agreed or agreed that they were confident in discussing HPV vaccination in their community. This increased to 96% of the participants at post-program (*p* < 0.001). We found similar sentiments when speaking to the CHWs in the focus groups. Several reported changes in their confidence due to the knowledge and resources that they gained in the ECHO.

*“I feel very confident because I have the resources…our instructor was super by giving us all the instructions and the resources and where to find them and how to bust the [HPV vaccination] myth(s). So, I felt when I left the ECHO very, very confident going back to the community and briefing it or giving it out.”*

*“If someone calls in with questions, I feel confident I can answer them or at least refer them resources.”*

The participants were more likely to view the HPV vaccine as cancer prevention. The participants who strongly agreed or agreed that the HPV vaccine is cancer prevention shifted from 84% pre-program to 95% post-program (*p* < 0.01); the largest movement was among those who selected strongly agree, which increased from 45% to 82%. The ability to focus on the vaccine as cancer prevention also emerged as a theme in the focus groups when the participants discussed how the ECHO program had impacted them.

*“And so just being able to learn to approach it [HPV vaccination] from the preventing cancer perspective and sharing it with my students in my class when we do talk about cancers and when we do talk about STDs…”*

*“I was not aware of all the different cancers that HPV vaccine can prevent, that was an eye opener for me.”*

The CHWs had desirable shifts in their vaccine beliefs about safety and sexual activity. A total of 80% of pre-program participants agreed that the HPV vaccine is safe compared to 97% post-program (*p* < 0.001). The proportion who agreed or were neutral that the HPV vaccine makes adolescents more likely to engage in sexual activity decreased from 29% to 12% (*p* < 0.001). We also saw a shift in belief around vaccination generally, with more participants strongly disagreeing that healthy children do not need vaccines post-program (97%) compared to pre-program (89%) (*p* < 0.05). 

### 3.4. Series Satisfaction, Usage, and Feedback

The post-survey feedback was overwhelmingly positive. The majority of participants strongly agreed or agreed that the ECHO was a good use of time (97.3%), the facilitation was organized and well done (97.3%), the instructor was prepared and knowledgeable (98.0%), and the content satisfying (98.0%). 

We found similar results in the focus groups. The CHWs found the content, resources, and strategies on how to address HPV vaccine myths very helpful. They also reported liking the trivia game used in the eighth session and the case presentations.

*“I really appreciated the very practical pieces especially helping us come up with the script and all the information we needed to give the people that we’re working with to inform them and educate them and help them reach that decision on using the HPV vaccine.”*

*“My favorite part was hearing each case presentation to see everyone in the class have an example on how they educated the people in the HPV vaccine through various teaching methods.”*

*“Everything was wonderful but the [case] presentations gave real life experiences. Session 8 was great with the Trivia [game].”*

Ninety-six percent strongly agreed or agreed that they would be able to apply skills derived from the ECHO as a CHW. The post-survey asked the participants how they planned to communicate about HPV with clients and community members in their everyday work, and responses included:

*“[I will] use [this information in] my setting at the school district to provide awareness.”*

*“I will be able to tell parents about the vaccine through routine calls.”*

*“All the time when I am talking with mothers or families in my education classes.”*

During the focus groups, three CHWs provided specific examples of successful interactions in their communities. These included talking with college students about HPV, having a child advocate for themself, and pre-COVID successes.

*“[I was] explaining [the HPV vaccine] to someone and then [was surprised] to have the actual child kind of tell the mom that they wanted to take it because they saw the importance of it.”*

When asked about the case presentations, 84.9% of the respondents agreed that they helped them apply information. The CHWs shared several suggestions in the focus groups for additional helpful resources including fliers, palm-sized pamphlets, ACS-branded materials, materials for people with low literacy levels, media resources, and video refreshers for CHWs.

## 4. Discussion

This manuscript is one of the first to detail the implementation and outcomes of a virtual HPV vaccination education program targeting community health workers. The evaluation results demonstrated that education using Project ECHO was an appropriate mechanism for increasing knowledge, beliefs, and confidence around the HPV vaccine. More research is needed on the impact of CHW community engagement on HPV vaccination uptake.

### 4.1. CHWs Have Potential to Drive HPV Vaccination Demand in Vulnerable Populations 

CHWs are frontline workers tasked with increasing public awareness and building the health of their community. They share lived experiences with those they serve and are often trusted leaders. CHWs have the potential to drive HPV vaccination demand through community education and navigation by addressing vaccine hesitancy and dis/misinformation and by reaching non-English speaking, vulnerable, or rural populations [14,50]. Educating CHWs with the Project ECHO model has proven to be a great strategy for the ACS’s HPVCFT Project to create HPV champions in Texas. Many participants in our program were themselves hesitant about the HPV vaccine. Increasing the confidence of CHWs is the first step in addressing community-level vaccine confidence. Additionally, CHWs may be able to help address the current deficit in HPV vaccine doses due to the pandemic, particularly for those adolescents receiving publicly funded doses through the Vaccines for Children (VFC) program. 

### 4.2. CHW Participants Demonstrated Increases in HPV Vaccination Knowledge

Like healthcare providers [51], we found that targeted virtual HPV vaccination education can improve the knowledge, beliefs, and confidence of CHWs. Both English and Spanish participants saw similar knowledge increases, suggesting that the programming was comparable across languages. 

The participants did not improve on the question assessing the recommended ages for HPV vaccination. We believe this confusion was due to the ACS changing its age recommendations partway through the implementation of the program [3]. The participants also scored poorly on the importance of provider recommendations on HPV vaccine uptake. The low performance on this question signals a gap in participant knowledge following the program. Existing evidence emphasizes the importance of provider recommendation on HPV vaccination uptake [52,53]. CHWs are ideally positioned to drive parents and adolescents to providers, but they need to clearly understand the importance of their role. Future efforts should provide CHWs with clear coaching on how to direct patients to vaccination providers. 

### 4.3. Participants Showed Desirable Shifts in HPV Vaccine Beliefs and Confidence Communicating

Addressing vaccine hesitancy and misinformation is a primary concern of healthcare providers and CHWs when talking to parents about the HPV vaccine [54,55]. Provider knowledge and beliefs about the vaccine have been shown to be associated with how they communicate the vaccine to patients [56]. Programs that work to change HPV vaccination knowledge and beliefs among healthcare workers are the first step to addressing community concerns and increasing uptake. Our program post-survey revealed positive shift in the foundational HPV vaccination beliefs of the CHW participants, most notably increasing their belief that the HPV vaccine is a cancer prevention vaccine. The CHWs also reported increased confidence in communicating about the HPV vaccine in their community, reaffirming the importance of HPV vaccination education that frames the HPV vaccine as cancer prevention and prepares CHWs to combat HPV myths. 

### 4.4. Implications for Linking CHWs to HPV Vaccination Opportunities

The participating CHWs were excited about the content and reported interest in using their HPV knowledge in the community. In the post-training focus groups, we found that some participants had a CHW certification but were employed in roles that did not involve more traditional CHW responsibilities like community education and outreach. Although Texas has a certification process and educational standards, there were wide variations in how our participants were embedded in the community, which will directly impact how they take action with what they learned in the program. These findings support the other literature on the widespread lack of standardization of CHW roles, responsibilities, and supervision [57,58].

Future research and interventions are needed to better understand how CHWs in the United States can be more systematically linked to vaccination opportunities or provided with clearer paths for directing patients to vaccination providers. Intervention, policy, or research opportunities to increase this linkage might include:

(1) We know that linking education to vaccination opportunities can increase vaccine uptake [18,59]. Are there ways to formalize the relationships between mobile clinics, school clinics, federally qualified health centers (FQHCs), or health departments and CHWs to better link HPV vaccine education in the community to vaccination opportunities?
(2) There is precedent for CHWs to be trained as vaccinators; in Alaska, CHWs administer vaccinations in rural communities [60]. What would it mean for trained Texan CHWs to administer vaccines themselves?
(3) We know that embedding CHWs into healthcare systems and clinical care teams can reduce healthcare costs and improve outcomes [58]. CHWs now have an option to bill for Medicaid rendered services as a part of the Cancer Moonshot effort [61,62]. Can CHWs be paid to increase linkages to doctors and clinics for vaccine visits following client interactions?
(4) CHWs were able to successfully support COVID-19 vaccination for vulnerable and hard-to-reach populations [29,61]. How could COVID-19 vaccination efforts with CHWs be adapted to HPV vaccination?
(5) CHWs in low- and middle-income countries (LMICs) often play a key role in primary care. Could this ECHO be adapted to LMICs with institutionalized CHW cadres where it is easier to assess the link between CHW education and vaccination uptake?

### 4.5. Strengths and Limitations

CHWs have established trust in communities and can reach vulnerable populations to educate them on HPV vaccination, address hesitancies, and combat health inequities in vaccination. This manuscript adds to the limited literature on HPV vaccination education programs for CHWs by detailing both the program implementation processes and outcomes. We offered this program numerous times in the first two years, reaching many CHWs in Texas and enabling us to collect meaningful quantitative and qualitative data on the program. There are several limitations to this study. We did not aim to generate generalizable knowledge on CHW knowledge, beliefs, and confidence about HPV vaccination, but instead to assess the changes resulting from this ECHO. Despite our best efforts, we saw attrition during the course, resulting in fewer post-surveys than pre-surveys. This could contribute to non-response bias, with our sample reflecting participants with higher course attendance or interest. Generally, we believe that our findings reflect the outcomes of those who attended, which is in line with our primary research question. We also experienced data quality issues with duplicate responses and missing CHW IDs that necessitated matching on the respondents’ entered names. Future efforts should aim to moderate attrition and data quality by shortening the course, requiring attendance at all sessions to receive CEs, or by transitioning to individualized links. Due to the volume of the course offerings, we did not have the capacity for 6-month follow-up surveys or to match the attendance information to an individual’s pre- and post-survey, limiting our understanding of knowledge retention and the impact of attendance on outcomes, which could be better explored in future research. Last, we were unable to directly assess how this program’s education for CHWs impacted HPV vaccination uptake due to the lack of formal linkages CHWs have to the healthcare system. Future efforts should consider targeting CHWs employed directly by healthcare systems for education so that vaccination impacts might be measured. 

## Figures and Tables

**Table 1:** Evaluation planning matrix.

Evaluation Question	Metric(s)	Data Source(s)	Evaluation Domain
A. How useful was the course content?	5-point response scale from strongly disagree to strongly agree	Post-survey Focus groups	Process
1. The facilitation of this series was organized and well done.			
2. The CHW instructor was well prepared and knowledgeable.			
3. I am satisfied with the content covered in the ECHO clinic.			
4. The order of the presentation topics made sense to me.			
5. I trusted the information and feedback provided by the hub members.			
6. I will be able to apply skills from this ECHO as a CHW.			
B. How could the ECHO be improved?	Open-text response	Post-survey	Process
1. Tell us your favorite part of the HPV Vaccination ECHO.			
2. What would you change about the ECHO to improve it for future CHW?			
C. How many CHWs claimed CE credits?	Credits	TX DSHS	Output
D. How many CHWs participated?	Attendance	Zoom	Output
E. Did the ECHO increase HPV vaccination knowledge?	4 items, correct or incorrect	Pre-survey Post-survey	Outcome
1. How many doses of the HPV vaccine does the CDC recommend for adolescents? [Responses: a. 1, b. 2, c. 3, d. 2 or 3, depending on age *]			
2. At what ages does the CDC recommend for on time vaccination for HPV? [Responses: a. Ages 9 and 10, b. Ages 11 and 12 *, c. Ages 13 to 15, d. Ages 16 to 26]			
3. HPV can cause which of the following cancers? [Responses: a. Cervical, b. Throat, c. Penile, vaginal, vulvar, and anal, d. All of the above *			
4. Which of the following is recommended by the CDC as most important to increase HPV vaccination? [Responses: a. Parent education, b. Adolescent education, c. Strong provider recommendation *, d. Patient reminders]			
F. Did the ECHO change HPV vaccination attitudes, beliefs, and confidence of participants?	5-point response scale from strongly disagree to strongly agree	Pre-survey Post-survey	Outcome
1. I am confident discussing HPV vaccination in my community.			
2. HPV vaccination is cancer prevention.			
3. The HPV vaccine is safe.			
4. I believe the HPV vaccine makes adolescents more likely to engage in sexual activity.			
5. Healthy children do not need vaccinations.			

* Denotes a correct response.

**Table 2:** Participant demographics.

Variable, n (%) ^1^	English (n = 92)	Spanish (n = 54)	Total (n = 146)
**Age ^2^**	45.3 (13.0)	51.5 (11.0)	47.6 (12.6)
**Gender**			
Female	83 (90.2)	52 (96.3)	135 (92.5)
Male	9 (9.8)	0	9 (6.2)
Prefer not to respond	0	2 (3.7)	2 (1.4)
**Year of Participation**			
2020	37 (40.2)	32 (59.3)	69 (47.3)
2021	55 (59.8)	22 (40.7)	77 (52.7)
**Education Level**			
Advanced degree	14 (15.2)	2 (3.7)	16 (11.0)
Bachelor’s degree	33 (35.9)	10 (18.5)	43 (29.5)
Some college	21 (22.8)	11 (20.4)	32 (21.9)
High school/GED	14 (15.2)	25 (46.3)	39 (26.7)
Other	10 (10.9)	6 (11.1)	16 (11.0)
**Current CHW Employment Status**			
Full time	40 (43.5)	10 (18.5)	50 (34.2)
Part-time	10 (10.9)	10 (18.5)	20 (13.7)
Volunteer	9 (9.8)	19 (35.2)	28 (19.2)
Not employed as a CHW	33 (35.9)	15 (27.8)	48 (32.9)
**Time as a CHW ^3^**			
0–2 years	24 (40.7)	12 (30.8)	36 (36.7)
3–5 years	17 (28.8)	7 (18.0)	24 (24.5)
5–10 years	13 (22.0)	8 (20.5)	21 (21.4)
More than 10 years	5 (8.5)	12 (30.8)	17 (17.4)
**Employment Agency ^3^**			
Healthcare	17 (32.1)	5 (13.5)	22 (24.4)
Non-profit	21 (39.6)	16 (43.2)	37 (41.1)
Social Service Entity	3 (5.7)	3 (8.1)	6 (6.7)
University/academic	5 (9.4)	6 (16.2)	11 (22.2)
Other ^4^	7 (13.2)	7 (13.0)	14 (15.6)
**Role is at an organization that vaccinates ^3^**			
Yes	17 (28.85)	9 (23.1)	26 (26.5)
No	42 (71.2)	30 (76.9)	72 (73.5)

^1^ Numbers may not sum to 146 due to missing data and percentages may not sum to 100% due to rounding. ^2^ Continuous variables were reporting using mean (sd). ^3^ Excludes participants who reported not currently working as a CHW. ^4^ Other included schools, health plans, a church, and other organizations.

## Data Availability

The datasets presented in this article are not readily available due to privacy concerns. Requests to access deidentified datasets or evaluation tools should be directed to shaylen.foley@cancer.org. You can find up-to-date ACS HPV vaccination ECHO content at https://echo.cancer.org/priorities/hpv-vaccination/ (accessed on 22 May 2024) and additional HPV vaccination educational materials at https://www.cancer.org/cancer/risk-prevention/hpv/hpv-vaccine.html (accessed on 22 May 2024). You may direct additional requests or questions about educational decks or materials to interventions@cancer.org and echo@cancer.org.

## References

[1] CDC How Many Cancers Are Linked with HPV Each Year?. https://www.cdc.gov/cancer/hpv/statistics/cases.htm.

[2] CDC HPV Cancers Are Preventable. https://www.cdc.gov/hpv/hcp/protecting-patients.html.

[3] Saslow D., Andrews K.S., Manassaram-Baptiste D., Smith R.A., Fontham E.T.H. (2020). Human Papillomavirus Vaccination 2020 Guideline Update: American Cancer Society Guideline Adaptation. CA Cancer J. Clin..

[4] Pingali C. (2021). National, Regional, State, and Selected Local Area Vaccination Coverage Among Adolescents Aged 13–17 Years—United States, 2020. MMWR Morb. Mortal. Wkly. Rep..

[5] Kang Y., Meador S., Black C.L., Vogt T. (2024). Recovery of Measles-Containing and HPV Vaccine Ordering Post-COVID-19 Pandemic: Trends by Public vs. Private Funding Source, Urbanicity, and State—United States, January 2018–December 2022. Prev. Med..

[6] Villarroel M., Galinksy A., Lu P.-J., Pingali C., Valenzuela C. (2024). Human Papillomavirus Vaccination Coverage in Children Ages 9–17 Years: United States, 2022.

[7] Escoffery C., Riehman K., Watson L., Priess A.S., Borne M.F., Halpin S.N., Rhiness C., Wiggins E., Kegler M.C. (2019). Facilitators and Barriers to the Implementation of the HPV VACs (Vaccinate Adolescents Against Cancers) Program: A Consolidated Framework for Implementation Research Analysis. Prev. Chronic. Dis..

[8] Fisher-Borne M., Preiss A.J., Black M., Roberts K., Saslow D. (2018). Early Outcomes of a Multilevel Human Papillomavirus Vaccination Pilot Intervention in Federally Qualified Health Centers. Acad. Pediatr..

[9] Foley S., Nkonga J., Fisher-Borne M. (2023). Engaging Health Plans to Prioritize HPV Vaccination and Initiate at Age 9. Hum. Vaccines Immunother..

[10] Isher-Witt J., Foley S., Hassan A., Sloan A., Nkonga J., Fisher-Borne M. (2023). Age Nine Is Possible: Improving Age 9 HPV Initiation through a National Quality Improvement Initiative during the COVID-19 Pandemic. Hum. Vaccines Immunother..

[11] Perkins R.B., Foley S., Hassan A., Jansen E., Preiss S., Isher-Witt J., Fisher-Borne M. (2021). Impact of a Multilevel Quality Improvement Intervention Using National Partnerships on Human Papillomavirus Vaccination Rates. Acad. Pediatr..

[12] (2021). 2021 Annual Report.

[13] Horner S.D., Fouladi R.T. (2008). Improvement of Rural Children’s Asthma Self-Management By Lay Health Educators. J. Sch. Health.

[14] Logan R.I., Castañeda H. (2020). Addressing Health Disparities in the Rural United States: Advocacy as Caregiving among Community Health Workers and Promotores de Salud. Int. J. Environ. Res. Public. Health.

[15] Andreae S.J., Andreae L.J., Cherrington A.L., Lewis M., Johnson E., Clark D., Safford M.M. (2018). Development of a Community Health Worker Delivered Cognitive Behavioral Training Intervention for Individuals with Diabetes and Chronic Pain. Fam. Community Health.

[16] Balcazar H., Rosenthal E.L., Brownstein J.N., Rush C.H., Matos S., Hernandez L. (2011). Community Health Workers Can Be a Public Health Force for Change in the United States: Three Actions for a New Paradigm. Am. J. Public Health.

[17] Kim K., Choi J.S., Choi E., Nieman C.L., Joo J.H., Lin F.R., Gitlin L.N., Han H.-R. (2016). Effects of Community-Based Health Worker Interventions to Improve Chronic Disease Management and Care Among Vulnerable Populations: A Systematic Review. Am. J. Public Health.

[18] Knowles M., Crowley A.P., Vasan A., Kangovi S. (2023). Community Health Worker Integration with and Effectiveness in Health Care and Public Health in the United States. Annu. Rev. Public Health.

[19] Sabo S., Allen C.G., Sutkowi K., Wennerstrom A. (2017). Community Health Workers in the United States: Challenges in Identifying, Surveying, and Supporting the Workforce. Am. J. Public Health.

[20] Kangovi S., Mitra N., Grande D., Huo H., Smith R.A., Long J.A. (2017). Community Health Worker Support for Disadvantaged Patients With Multiple Chronic Diseases: A Randomized Clinical Trial. Am. J. Public Health.

[21] Palmas W., March D., Darakjy S., Findley S.E., Teresi J., Carrasquillo O., Luchsinger J.A. (2015). Community Health Worker Interventions to Improve Glycemic Control in People with Diabetes: A Systematic Review and Meta-Analysis. J. Gen. Intern. Med..

[22] Wells K.J., Luque J.S., Miladinovic B., Vargas N., Asvat Y., Roetzheim R.G., Kumar A. (2011). Do Community Health Worker Interventions Improve Rates of Screening Mammography in the United States? A Systematic Review. Cancer Epidemiol. Biomarkers Prev..

[23] Fernandez M.E., Savas L.S., Lipizzi E., Smith J.S., Vernon S.W. (2014). Cervical Cancer Control for Hispanic Women in Texas: Strategies from Research and Practice. Gynecol. Oncol..

[24] Fleming K., Simmons V.N., Christy S.M., Sutton S.K., Romo M., Luque J.S., Wells K.J., Gwede C.K., Meade C.D. (2018). Educating Hispanic Women about Cervical Cancer Prevention: Feasibility of a Promotora-Led Charla Intervention in a Farmworker Community. Ethn. Dis..

[25] Parra-Medina D., Morales-Campos D.Y., Mojica C., Ramirez A.G. (2015). Promotora Outreach, Education and Navigation Support for HPV Vaccination to Hispanic Women with Unvaccinated Daughters. J. Cancer Educ. Off. J. Am. Assoc. Cancer Educ..

[26] Elam-Evans L.D. (2020). National, Regional, State, and Selected Local Area Vaccination Coverage Among Adolescents Aged 13–17 Years—United States, 2019. MMWR Morb. Mortal. Wkly. Rep..

[27] About the ECHO Model. https://hsc.unm.edu/echo/what-we-do/about-the-echo-model.html.

[28] Bouchonville M.F., Hager B.W., Kirk J.B., Qualls C.R., Arora S. (2018). Endo echo improves primary care provider and community health worker self-efficacy in complex diabetes management in medically underserved communities. Endocr. Pract. Off. J. Am. Coll. Endocrinol. Am. Assoc. Clin. Endocrinol..

[29] Damian A.J., Robinson S., Manzoor F., Lamb M., Rojas A., Porto A., Anderson D. (2020). A Mixed Methods Evaluation of the Feasibility, Acceptability, and Impact of a Pilot Project ECHO for Community Health Workers (CHWs). Pilot Feasibility Stud..

[30] Recto P., Lesser J., Zapata J., Moreno-Vasquez A., Gandara E., Zavala Idar A., Castilla M. (2022). The Development and Implementation of a COVID-19 Project ECHO: A Program for Community Health Workers Serving Populations from Rural and Medically Underserved Areas in South Texas. Issues Ment. Health Nurs..

[31] Thomas K.T., Friedman S.A., Larson M.J., Jorgensen T.C., Sharma S., Smith A., Lavi M.S. (2023). A Cohort-Based Nutrition ECHO for Community Health Workers. Health Educ. Res..

[32] Lopez M.S., Baker E.S., Milbourne A.M., Gowen R.M., Rodriguez A.M., Lorenzoni C., Mwaba C., Msadabwe S.C., Tavares J.H., Fontes-Cintra G. (2017). Project ECHO: A Telementoring Program for Cervical Cancer Prevention and Treatment in Low-Resource Settings. J. Glob. Oncol..

[33] Salcedo M.P., Gowen R., Lopez M., Baker E., Rodriguez A.M., Milbourne A., Fisher-Hoch S., Ogburn T., Daheri M., Guerra L.B. (2019). Addressing the High Cervical Cancer Rates along the Texas-Mexico Border through Community Outreach, Patient Navigation, and Provider Training/Telementoring. Gynecol. Oncol..

[34] (2016). Zoom Version 5.

[35] Centers for Disease Control and Prevention (1999). Framework for Program Evaluation in Public Health. MMWR.

[36] (2020). SurveyMonkey Premier.

[37] Malo T.L., Hall M.E., Brewer N.T., Lathren C.R., Gilkey M.B. (2018). Why Is Announcement Training More Effective than Conversation Training for Introducing HPV Vaccination? A Theory-Based Investigation. Implement. Sci..

[38] Fay M.P., Hunsberger S.A., Nason M., Gabriel E., Lumbard K. (2023). Exact2×2: Exact Tests and Confidence Intervals for 2 × 2 Tables. https://cran.r-project.org/web/packages/exact2x2/exact2x2.pdf.

[39] R Core Team (2013). R: A Language and Environment for Statistical Computing.

[40] RStudio Team (2020). RStudio: Integrated Development Environment for R.

[41] StataCorp (2021). Stata Technical Support.

[42] Wickham H., Averick M., Bryan J., Chang W., McGowan L.D., François R., Grolemund G., Hayes A., Henry L., Hester J. (2019). Welcome to the Tidyverse. J. Open Source Softw..

[43] Kuhn M., Wickham H. (2020). Tidymodels: A Collection of Packages for Modeling and Machine Learning Using Tidyverse Principles. https://www.tidymodels.org.

[44] Iannone R., Cheng J., Schloerke B., Hughes E., Seo J. (2022). Gt: Easily Create Presentation-Ready Display Tables. https://cran.r-project.org/web/packages/gt/index.html.

[45] Slowikowski K. (2023). Ggrepel: Automatically Position Non-Overlapping Text Labels with ggplot2. https://rdrr.io/cran/ggrepel/.

[46] Wilke C.O., Wiernik B.M. (2022). Ggtext: Improved Text Rendering Support for ggplot2. https://wilkelab.org/ggtext/.

[47] Auguie B. (2017). gridExtra: Miscellaneous Functions for “Grid” Graphics. https://cran.r-project.org/web/packages/gridExtra/index.html.

[48] (2022). VERBI Software MAXQDA. https://www.maxqda.com/about.

[49] Rev Transcription Services 2022. https://www.rev.com/.

[50] Brownstein J.N., Allen C.G. (2015). Addressing Chronic Disease through Community Health Workers: A Policy and Systems-Level Approach.

[51] Leung S.O.A., Akinwunmi B., Elias K.M., Feldman S. (2019). Educating Healthcare Providers to Increase Human Papillomavirus (HPV) Vaccination Rates: A Qualitative Systematic Review. Vaccine X.

[52] Gilkey M.B., McRee A.-L. (2016). Provider Communication about HPV Vaccination: A Systematic Review. Hum. Vaccines Immunother..

[53] Perkins R.B., Zisblatt L., Legler A., Trucks E., Hanchate A., Gorin S.S. (2015). Effectiveness of a Provider-Focused Intervention to Improve HPV Vaccination Rates in Boys and Girls. Vaccine.

[54] Moya E.M., Garcia A., Joyce Ponder A., Frietze G. (2023). Addressing Knowledge Gaps: The Key Role of Community Health Workers and Healthcare Providers in Human Papillomavirus Prevention and Vaccine Uptake in a Border Community. Front. Public Health.

[55] Zimet G.D., Rosberger Z., Fisher W.A., Perez S., Stupiansky N.W. (2013). Beliefs, Behaviors and HPV Vaccine: Correcting the Myths and the Misinformation. Prev. Med..

[56] Rodriguez S.A., Mullen P.D., Lopez D.M., Savas L.S., Fernández M.E. (2020). Factors Associated with Adolescent HPV Vaccination in the U.S.: A Systematic Review of Reviews and Multilevel Framework to Inform Intervention Development. Prev. Med..

[57] Brown O., Kangovi S., Wiggins N., Alvarado C.S. (2020). Supervision Strategies and Community Health Worker Effectiveness in Health Care Settings.

[58] Pinto D., Carroll-Scott A., Christmas T., Heidig M., Turchi R. (2020). Community Health Workers: Improving Population Health through Integration into Healthcare Systems. Curr. Opin. Pediatr..

[59] Kaul S., Do T.Q.N., Hsu E., Schmeler K.M., Montealegre J.R., Rodriguez A.M. (2019). School-Based Human Papillomavirus Vaccination Program for Increasing Vaccine Uptake in an Underserved Area in Texas. Papillomavirus Res..

[60] Gibson E., Zameer M., Alban R., Kouwanou L.M. (2023). Community Health Workers as Vaccinators: A Rapid Review of the Global Landscape, 2000–2021. Glob. Health Sci. Pract..

[61] Cancer Moonshot. https://www.whitehouse.gov/cancermoonshot/.

[62] Centers for Medicare & Medicaid Services (2023). CMS Physician Payment Rule Advances Health Equity.

